# RACIAL DIFFERENCES IN LOCATION AND ACTIVITY-SPECIFIC FALLS IN MASSACHUSETTS, US

**DOI:** 10.1093/geroni/igac059.420

**Published:** 2022-12-20

**Authors:** Wenjun Li, Linda Churchill, Annabella Aguirre, Anthony Clarke, Elizabeth Procter-Gray, Kevin Kane, Sarah Berry, Marian Hannan

**Affiliations:** University of Massachusetts Lowell, Lowell, Massachusetts, United States; University of Massachusetts Lowell, Lowell, Massachusetts, United States; University of Massachusetts Lowell, Lowell, Massachusetts, United States; University of Massachusetts Lowell, Lowell, Massachusetts, United States; University of Massachusetts Lowell, Lowell, Massachusetts, United States; University of Massachusetts Lowell, Lowell, Massachusetts, United States; Hebrew SeniorLife / Harvard Medical School, Boston, Massachusetts, United States; Hebrew SeniorLife, Roslindale, Massachusetts, United States

## Abstract

Prevention of falls is important to independent living and good health in older age. Circumstances of falls may vary by older person’s sociodemographic attributes, health conditions and living environment. Health Aging and Neighborhood Study (HANS) examined rates of location and activity-specific falls among 388 community-living older adults living in Central Massachusetts between 2018 and 2020. Data on falls were collected using monthly calendar, and information on falls was collected by trained staff via telephone interviews with the participants. The follow-up time ranging from 11.9 to 24.4 months. In total, 431 falls were reported, of which 97% had information on location of fall, and 95% had information on activity at time of fall. Annualized rate of any fall differed significantly by race/ethnicity. Asians had the lowest rate (0.26/year). Rate of Hispanics was approximately twice higher (0.55, p=0.04) and non-Hispanic Whites three times higher (0.84, p<0.001). For indoor falls, Asians reported significantly lower rates than Hispanics (0.10 vs. 0.44, p=0.005) or non-Hispanic Whites (0.10 vs. 0.36, p=0.009). Greater outdoor fall rate was associated with male gender (p=0.04); younger age (p=0.03); Asian (p=0.02) and, especially, non-Hispanic White race (p<0.001, with Hispanics as referent); higher income (p<0.001); and more years of education (p=0.001). Falls while walking were significantly associated with non-Hispanic White race (p<0.001) and Hispanic ethnicity (p=0.001). In summary, rates of any, indoor or outdoor falls vary significantly by race. The underlying mechanisms should be further investigated.

